# Supplementary material for: Effects of Titanium–Silica Oxide on Degradation Behavior and Antimicrobial Activity of Poly (Lactic Acid) Composites
Source: Polymers (Basel). 2022 Aug 14;14(16):3310. doi: 10.3390/polym14163310 (PMC9416649; doi:10.3390/polym14163310)
Supplement: Supplementary file 1 [file polymers-14-03310-s001.zip › polymers-1840281-supplementary.pdf]

## 1. Results and discussion section

(a)

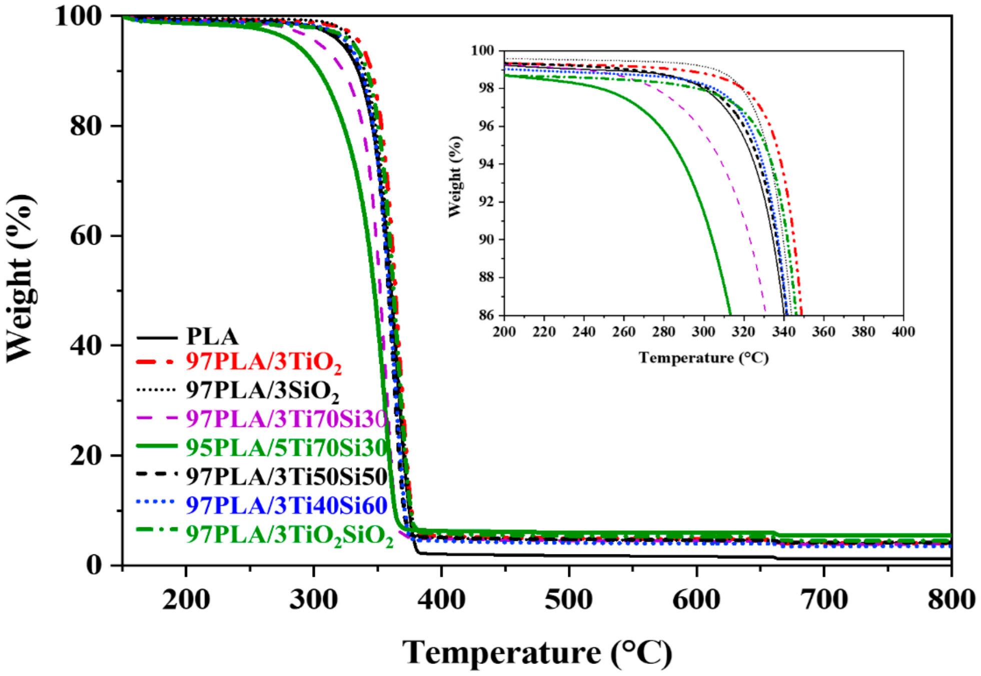


(b)

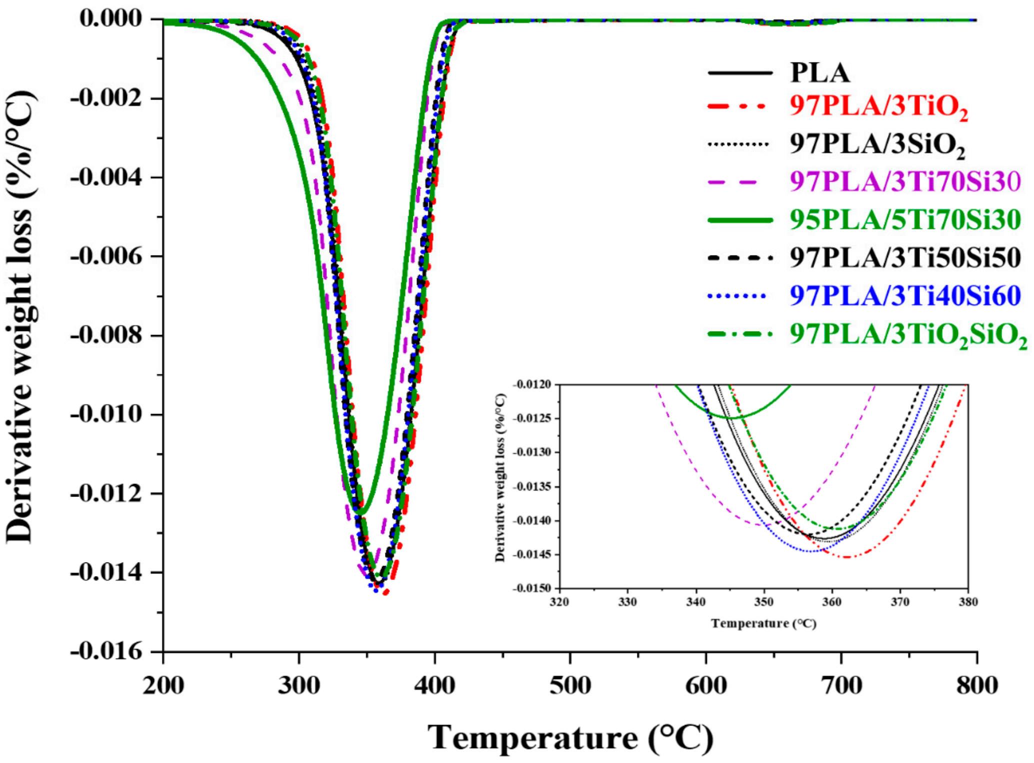


**Figure s1.** Curve of (a) TGA thermogram (b) DTG Thermogram of PLA, PLA/$TiO_2$, PLA/$SiO_2$, PLA/$Ti_xSi_y$, and PLA/$TiO_2SiO_2$ composites.

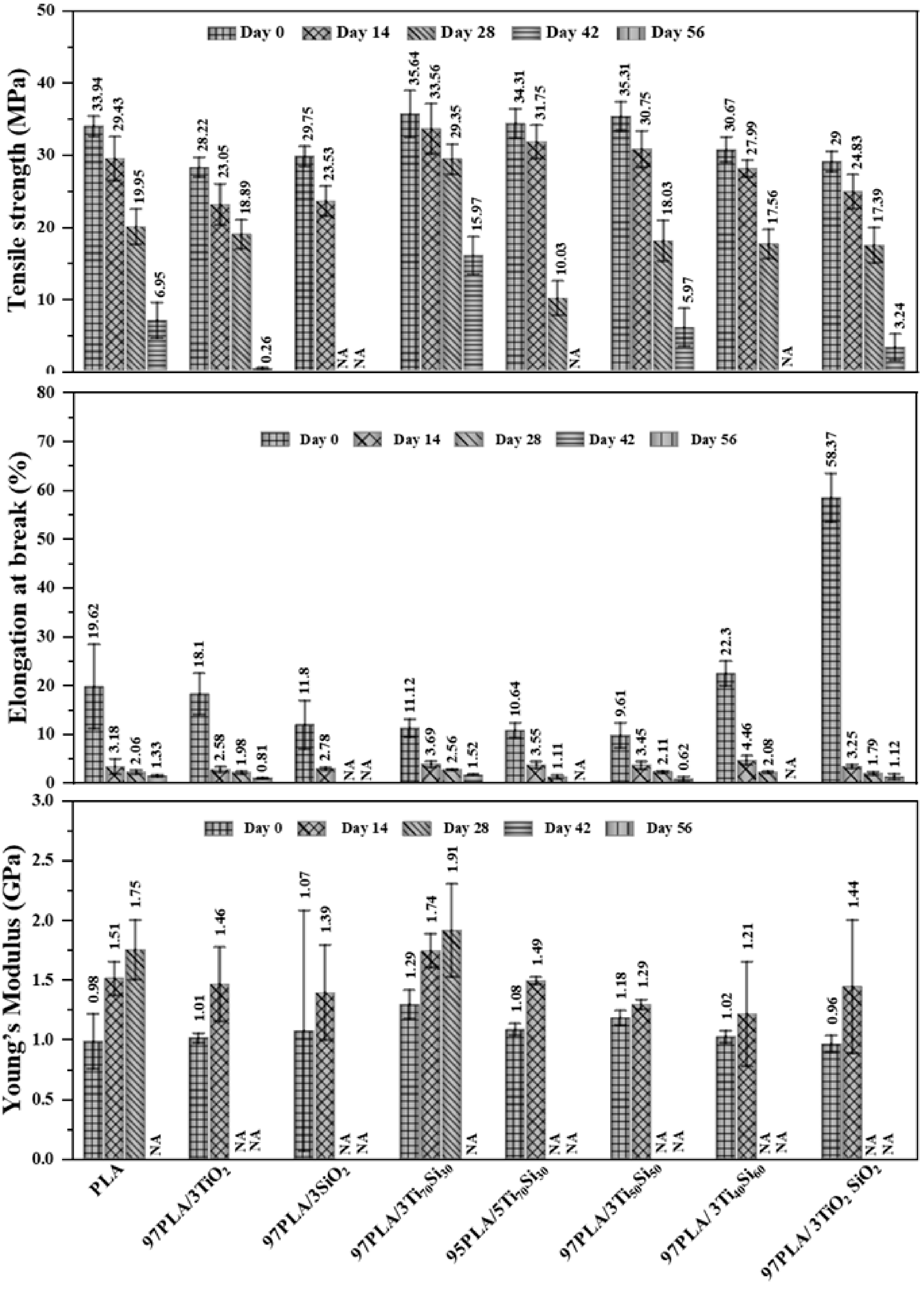


**Figure s2.** The changes in tensile properties of PLA, PLA/$TiO_2$, PLA/$SiO_2$ and PLA/$Ti_xSi_y$ composite films after different period of *in vitro* degradation.
